# Secondary prevention of cardiovascular disease: is it time for the polypill to be standard treatment?

**DOI:** 10.1016/j.lanepe.2025.101384

**Published:** 2025-07-12

**Authors:** Tom Marshall

**Affiliations:** Department of Applied Health Sciences, University of Birmingham, Edgbaston, Birmingham, B15 2TT, UK

Fixed-dose combination therapy (the polypill) was first proposed for cardiovascular prevention over two decades ago.^1^ While fixed-dose combination therapy has long been accepted in the treatment of HIV and tuberculosis, adoption has been slow for cardiovascular prevention. Evidence favouring polypill strategies has gradually accumulated, the paper by Gaziano et al. in this issue of The Lancet Regional Health–Europe paper is a useful addition to the evidence.^2^

There are many variants of the polypill, but most include antihypertensives, statins and lipid-lowering drugs. Adoption of a polypill has faced institutional challenges.^3^ Regulatory processes are designed for new, single drugs and clinical teaching has tended to emphasise initiation of drugs sequentially. There are limited incentives for pharmaceutical companies to invest in developing a polypill as the components are generic.

Originally conceived as a simplified, universal, primary prevention strategy for anyone at high risk of cardiovascular disease, this was undoubtedly a revolutionary proposal. But this radical suggestion may have distracted from the more prosaic advantages of a polypill in secondary prevention–patients with existing cardiovascular disease. There has generally been greater openness to the polypill for both primary and secondary prevention in lower and middle-income countries and in underserved populations in high income countries.^4^, ^5^, ^6^ The practicality of the approach is seen as its principal virtue. But this also has its drawbacks. The focus on a polypill in less well-resourced settings may have given the impression that it is a second-best strategy in comparison to usual care in high-income countries. Such an impression would be misleading.

The three component polypill developed by the Spanish National Centre for Cardiovascular Research (CNIC) has overcome some of the regulatory challenges. Firstly, it is intended for secondary prevention after myocardial infarction. Secondly, it includes only three drugs—an antiplatelet agent, antihypertensive and a lipid-lowering drug—which are standard treatment in secondary prevention after a myocardial infarction. Much of the rationale for the polypill strategy is its simplicity: combining treatments into a single tablet facilitates treatment initiation and medication adherence. This simplicity is known to appeal to patients, who prefer to take fewer tablets.^7^ As well as addressing therapeutic inertia, by reducing the number of decisions to be taken, fixed-dose combinations also reduce the temptation to tamper with treatments in response to monitoring. Both blood pressure and lipid levels vary within-individuals, even apparently clinically important changes between measurements are indistinguishable chance variation.^8^^,^^9^

This paper reports the cost-effectiveness of a strategy for secondary prevention of cardiovascular disease using a fixed-dose combination therapy of aspirin, atorvastatin and ramipril compared to usual care. The setting is high-income European countries, with universal access to healthcare. The combination was previously shown to be more effective than usual care at reducing cardiovascular events, likely mediated through higher medication adherence.^10^ This paper reports the cost-effectiveness of the strategy compared to usual care. The modelling includes some minor simplifications: ignoring second and subsequent cardiovascular events and ignoring possible small effects on incidence of diabetes.^2^ On balance these might be expected to underestimate the cost-effectiveness of the polypill strategy. Nevertheless, under 84·8% of scenarios, it was more effective and cost less than usual care. In short, this is a better, cheaper strategy, even in countries where patients have access to a high standard of care.

The cost-effectiveness analysis makes a strong case for secondary cardiovascular prevention after myocardial infarction using this three component polypill. It seems hard to argue that fixed-dose combination should not be the standard treatment for secondary prevention after myocardial infarction. Why would we use a less effective and more costly approach to treatment? This does not mean all questions have been answered. Further research should investigate the effectiveness and cost-effectiveness of polypills for secondary prevention in patients with other types of cardiovascular diseases. Research could also investigate whether more intensive lipid lowering and blood pressure lowering with higher doses of statins and two or three antihypertensive drugs might be more effective than the combination used in this clinical trial. A comparison between usual care and a polypill for primary prevention in patients at high predicted risk of cardiovascular disease would also help address further questions about the polypill strategy.

## Contributors

Tom Marshall.

## Declaration of interests

The author has no competing interests.
